# What actually happens in partnered health research? A concordance analysis of agreement on partnership practices in funded Canadian projects between academic and knowledge user investigators

**DOI:** 10.1186/s12874-025-02679-y

**Published:** 2025-10-06

**Authors:** Kathryn M. Sibley, Leah K. Crockett, Brenda Tittlemier, Ian D. Graham

**Affiliations:** 1 Department of Community Health Sciences, Rady Faculty of Health Sciences, University of Manitoba, Winnipeg, MB Canada; 2 George and Fay Yee Centre for Healthcare Innovation, 379-753 McDermot Avenue, Winnipeg, MB Canada; 3 Department of Pediatrics and Child Health, Rady Faculty of Health Sciences, University of Manitoba, Winnipeg, MB Canada; 4 Applied Health Sciences Program, University of Manitoba, Winnipeg, MB Canada; 5 Centre for Practice-Changing Research, Ottawa Hospital Research Institute, Ottawa, ON Canada; 6 School of Epidemiology and Public Health and School of Nursing, University of Ottawa, Ottawa, ON Canada

**Keywords:** Knowledge users, Health research, Partnership practices, Partnered research

## Abstract

**Background:**

Collaborations involving partnerships between academic researchers and knowledge users can improve the relevance and potential adoption of evidence in health care practices and decision-making. However, descriptions of partnering practice characteristics are often limited to self-report from the lead academic researcher, with no comparison among team members. The primary objective of this study was to determine the extent to which nominated principal investigator (NPI) respondents of a questionnaire about funded Canadian partnered health research projects agreed with other team researchers and knowledge users (KU) on partnership practices.

**Methods:**

We conducted secondary analysis of a subset of data from 106 respondents from 53 partnered Canadian health research projects funded between 2011 and 2019. We organized projects into NPI-researcher and NPI-KU dyads, and analyzed 23 binary variables about types of knowledge users involved and approaches for involving knowledge users in the project. We calculated Kappa scores and examined if agreement varied by dyad type and time across three blocks of years of project funding using a two-way ANOVA. We also explored how agreement varied by question type (independent t-test) and by variable (Pearson Chi-Square).

**Results:**

Overall agreement on partnership practices was minimal (mean Kappa = 0.38, SD 0.27). NPI- researcher dyads had higher Kappa scores than NPI-KU dyads (*p* = 0.03). There were no significant differences across funding year blocks (*p* > 0.05). Agreement on the types of knowledge users engaged in the project was weak (mean Kappa = 0.43, SD 0.32), and there was no difference by dyad type. Agreement was minimal on the approaches for involving knowledge users the project (mean Kappa = 0.28, SD 0.31), and NPI-researcher dyads had significantly higher Kappa scores than NPI-KU dyads (*p* = 0.03). Variable-level agreement ranged between 47 and 98%.

**Conclusions:**

The overall low level of agreement among team members responding about the same project has implications for the continued study and practice of partnered health research. These findings highlight the caution that must be used in interpreting retrospectively assessed self-report practices. Moving forward, prospective documentation of partnered research practices offers the greatest potential to overcome the limitations of recall-based retrospective analyses.

## Background

Health research is highly collaborative, often involving teams of two or more individuals with complementary expertise who interact to undertake specific tasks to achieve research goals [1]. While such teams have classically included individuals from many academic backgrounds, there has been growth in the inclusion of individuals from health care and health systems, community representatives, and people with lived experience of a health condition (among others) as formal members of research teams. These individuals are often referred to as knowledge users, and their involvement has been positioned as a mechanism for health research to better address issues affecting practice and support application of health research evidence, relative to the longstanding academically driven approaches to research [2, 3]. The nature of and expectations for knowledge user involvement can vary substantially, though there is consensus on the need for meaningful involvement that avoids tokenism [4]. Such involvement could be enabled through many aspects of a project and how a team interacts in practice, shifting from consultation and information sharing towards shared decision-making and leadership [5]. The acknowledged potential of this specific form of research collaboration has been promoted and incentivized by numerous health research funding organizations [6, 7], where formal involvement with knowledge users as team members is recognized, and at times required, in external funding for health research projects and/or programs. For example, in Canada, both federal and provincial health research funding agencies have formally included calls for partnered research with knowledge users as strategy to enhance relevance and uptake of health research evidence [6].

However, despite a growing body of literature examining many aspects of health research involving knowledge users [8, 9], there remains limited evidence on key issues pertaining to mechanisms of action in partnered health research teams and their relationship to outcomes and effects. While some of these limitations are not unique to research teams involving knowledge users and may apply in studies of team science more broadly, the unique aspects of health research conducted in partnership with knowledge users, such as the potential for power disparities, varying valuation of differing knowledge systems, and risks of tokenism [10], make it an important area of continued study.

These limitations in evidence are due in part to numerous challenges that exist in studying these teams that affect the nature and quality of data collected and the conclusions that can be drawn from them. For example, many quantitative studies of partnered research rely on self-report from a single individual representing the project [such as [11, 12]. This is often the lead academic team member, who is assumed to be the most reliable informant about what happened on a team/project because they typically initiate and direct the work and dedicate the most time on the project. However, there is a risk that data provided by a single informant pertaining to the characteristics, outcomes, and effects of a project may be interpreted erroneously as objective “facts”, without triangulation or verification from other sources. While some studies have compared data between academic and knowledge user research team members [13], these have often been group comparisons using qualitative approaches. We are not aware of any studies that have quantitatively analyzed such “facts”, i.e. such as practices undertaken in partnering, or compared variations in self-report from team members from the same project or collaboration [14–16].

In 2020, 589 individuals named in leadership roles from 456 partnered health research projects funded in Canada between 2011 and 2019 completed a questionnaire about collaborative research practices and perceived effects of involving knowledge users in the eligible project [17]. Respondents included academic researchers working in administrative leadership roles on the eligible project [referred to as nominated principal investigators (NPI)], academic researcher team members, and knowledge user (KU) team members. Our sampling approach facilitated responses from multiple team members on eligible projects, presenting a unique opportunity for data analysis. Of the 456 projects in the data set, 53 included responses from the NPI and at least one other team member.

Data from respondents from the same research project presents an opportunity for examination of the level of agreement, i.e., concordance, between research team members. Research on partnership practices, i.e., the features and activities undertaken, has included either the reports of academic researchers or knowledge users but not as matched pairs that examined the extent of their agreement on the practices. Accurately determining partnership practices is a critical precursor for understanding meaningful, effective, and impactful involvement of knowledge users in health research. Accordingly, understanding the extent of agreement when multiple respondents answer the same questions about a research project is important because it provides an indication of data reliability and an indication of the representativeness of a single respondents’ answers about a project. Our primary objective in this analysis was to determine and compare the extent to which KU and researcher team members’ responses about partnership practices in the eligible project agreed with the NPI (overall agreement). Our secondary objectives were to explore if agreement varied by time since funding, and to explore and compare agreement within questions and variables.

## Methods

### Context and conceptual foundations

This study is a component of a set of research projects to understand practices in Canadian partnered health research and develop practice recommendations (CIHR grant PJT # 156372). The contexts and conceptual framework that guided this work have been described elsewhere [18]. In brief, a pragmatic approach using integrated knowledge translation [19] has underpinned the projects.

### Study design

Secondary analysis of data from a cross-sectional survey [17].

### Data source

Details of sampling and the survey have been previously reported [17, 18]. In brief, we identified 1153 eligible partnered health research projects funded between 2011 and 2019. The eligible projects all received peer-reviewed funding from a federal or provincial health research funding agency and included knowledge users on the project team (through a funding requirement, reporting knowledge user involvement, or using a collaborative research approach such as integrated knowledge translation, community-based participatory research, or patient-oriented research) [18]. We included projects from funding opportunities that required a non-academic partner, reported involving research users on the research team or identified as using a collaborative research approach (e.g., iKT, CBPR or POR) at the time of application. Projects were identified between fall 2019 and winter 2020. Projects spanned a range of health research topics (137 fields of research identified) and were categorized as health and social care services research [17]. We identified and invited 2155 individuals named as investigators on these projects using a modified Dillman approach. We invited all principal investigators (both researchers and knowledge users), and in cases of only one principal investigator we also invited co-investigators. Individuals with more than one project were invited to respond to the oldest eligible project. The full project team, which included people with lived experience of a health condition, people with lived professional experience (including health professionals and knowledge translation practitioners), community organization and research funding organization representatives, trainees and academic researchers who practice and/or study health research collaborations with knowledge users, developed a questionnaire that explored numerous aspects of the eligible research project, including respondent role and project details, respondent characteristics, partnership practices, perceived effects of involving knowledge users in the project, as well as team cohesion, individual capabilities, opportunities and motivations for working in partnership, and knowledge user experiences. The questionnaire was pilot tested with 12 individuals and revised iteratively until no further feedback was received. Data were collected between August- October 2020. Of the 742 responses (34% of invitees), data from 589 respondents (92% of those eligible) reporting on 456 projects were included in the primary analysis. Respondents were composed of 42% NPIs, 40% academic researcher team members, and 18% KUs.

In this study, we included 23 variables on practices undertaken in the eligible partnered research project from two questions (Table 1). We selected practice variables only because they represented a characteristic of the project that we hypothesized should be the same across project team members, rather than a respondent perception, which we expected to vary between individuals or questions pertaining to individual reflections. These questions asked about types of knowledge users engaged in the project (select all that apply format, nine binary variables with an “I don’t know” option) and approaches used to engage knowledge users (select all that apply format, 14 binary variables with an “I don’t know” option). For six dyads, one respondent selected “I don’t know” to one of the questions, or did not provide a response, which meant kappa scores could not be calculated for one of the two questions. To retain the maximum usable data, concordance was calculated for the question with substantive response. We categorized each project into three-year blocks by year of funding (2011–2013, 2014–2016, 2017–2019) to explore temporal effects on agreement levels. We analyzed data from 53 dyads that included data from the NPI and a researcher or KU team member of an eligible project (106 respondents). In projects with multiple team members in addition to the NPI (*n* = 15), we limited to one dyad per project, prioritized selecting an NPI-KU dyad and used random selection if there were multiple dyads.

**Table 1:** Variables included in the analysis

Question	Response Option (Binary Variable)
Which knowledge users are/were involved in this project?	a. Person with lived experience of a health condition b. Community member c. Health professional d. Healthcare manager or administrator e. Health system decision or policy maker f. Community organization representative g. Health research funding organization representative h. Health professional organization representative i. Industry representative
Which approaches or activities are/were used for involving knowledge users in this project?	a. Formal in-person research project meeting(s)- individual and/or group b. Formal online research project meeting(s)- individual and/or group c. Formal updates or newsletters about the research project- electronic and/or hard copy d. Informal conversations – in-person, telephone, and/or electronic e. Distribution of study documents f. Shared electronic space for saving research project documents g. Development of formal documentation or process h. Establishment of formal working groups i. Provision of social opportunities j. Provision of training opportunities and/or resource materials k. Honorarium for research users l. Reimbursement of expenses for research users m. Sharing of research funds with research user organization n. Researchers attending research user meetings or events

### Data analysis

All analyses were performed using SPSS © version 28. We determined the degree of agreement between respondents from the same project with the Kappa statistic [20]. We calculated Kappa scores across the 23 variables to calculate a measure of overall within-project agreement. We used descriptive statistics to summarize the distribution and mean level of overall agreement across projects. We then compared mean overall Kappa scores by dyad type (NPI-researcher and NPI-KU) and funding year block using a two-way analysis of variance (ANOVA), with *p* < 0.05 indicating significance. We calculated a within-question Kappa score to determine agreement for (i) types of knowledge users engaged in the project and (ii) involvement activities undertaken in the project, and examined the effect of dyad type on agreement with an independent t-test. To explore within-variable agreement, we coded agreement for each variable in each project (agree, disagree, or missing), and then examined differences in the distribution of proportions with Pearson Chi-Square tests for each variable.

We interpreted Kappa values as follows: <0 (less than chance), 0-0.20 (none to slight agreement), 0.21–0.39 (minimal agreement), 0.40–0.59 (weak agreement), 0.60–0.79 (moderate agreement), 0.80–0.90 (strong agreement), and above 0.90 (almost perfect agreement) [21]. We considered Kappa values 0.60 and above as indicating acceptable agreement [21].

## Results

The 53 project dyads included 30 NPI-researcher dyads (57%) and 23 NPI-KU dyads (43%). Project characteristics are reported in Table 2, and respondent characteristics are reported in Table 3.

**Table 2:** Project dyad characteristics (*n* = 53)

	*N* (%) or mean (SD, range)
Funding type	
Federal	37 (70%)
Provincial	16 (30%)
Funding year block	
2011–2013	19 (36%)
2014–2016	18 (34%)
2017–2019	16 (30%)
Project funding amount (CAD) (*n* = 48)	375,789 (556319, 5000-3318872)
Project length (months) (*n* = 50)	27 (17, 2–84)
Number of named investigators	13 (10, 1–58)
Province of funded institution	
Ontario	12 (23%)
British Columbia	12 (23%)
Quebec	11 (21%)
Saskatchewan	8 (15%)
Manitoba	5 (9%)
Alberta	4 (8%)
Other provinces/territories	1 (2%)

**Table 3:** Respondent characteristics (*n* = 106)

		*N* (%)
Gender identity	Woman	59 (55%)
	Man	39 (37%)
	Prefer not to answer	3 (3%)
	Missing	5 (5%)
Member of a visible minority in Canada	Yes	9 (8%)
	No	90 (85%)
	Prefer not to answer	2 (2%)
	Missing	5 (5%)
Indigenous	Yes	3 (3%)
	No	96 (90%)
	Prefer not to answer	2 (2%)
	Missing	5 (5%)
Project role	Nominated principal investigator researcher	53 (50%)
	Other researcher	30 (28%)
	Knowledge user	23 (22%)
	Health system decision or policy maker	5 (22%)
	Healthcare manager or administrator	7 (30%)
	Community organization representative	2 (9%)
	Health professional	2 (9%)
	Technical or research support professional	2 (9%)
	Health professional organization representative	1 (4%)
	Person with lived experience of health condition	2 (9%)
	Knowledge translation professional	1 (4%)
	Health research funding organization representative	1 (4%)

### Overall agreement on partnership practices

**Fig. 1:**
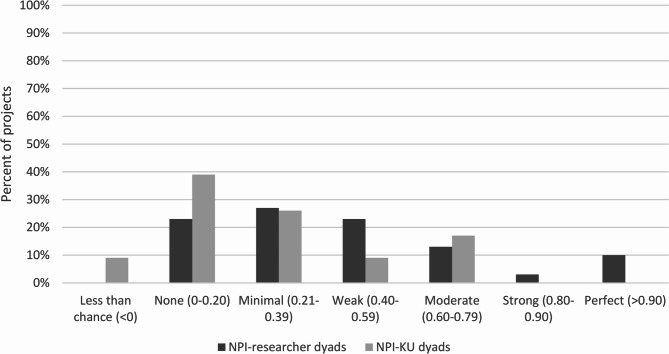
Overall Kappa distribution by dyad type

Overall agreement on partnership practices within a project was minimal (mean Kappa = 0.38, SD = 0.27, range= −0.11–1.00). Figure 1 illustrates the distribution of overall Kappa scores. Twelve projects (22.6%) had acceptable agreement. There was no significant interaction between dyad type and funding year block. There was a significant main effect of dyad type on overall agreement [F(1,47) = 4.74, *p* = 0.03]. NPI-team researcher dyads had higher Kappa scores (mean Kappa = 0.45, SD = 0.26) than NPI-KU dyads (mean Kappa = 0.28, SD = 0.25). There was no significant effect of funding year block.

### Within-question agreement

Agreement on the types of knowledge users engaged in the project was weak (mean Kappa = 0.43, SD = 0.32), and there was no difference by dyad type. Agreement was minimal on the involvement activities undertaken in the project (mean Kappa = 0.28, SD = 0.31). There was a significant difference between dyad type on agreement in involvement activities [t(45) = 2.26, *p* = 0.03]. NPI-researcher dyads had significantly higher Kappa scores (mean Kappa = 0.37, SD = 0.31) than NPI-KU dyads (mean Kappa = 0.18, SD = 0.27).

### Within-variable agreement (Table 4)

Agreement on the variables of knowledge users engaged in the project ranged between 47.2 and 98.1%. Industry representative involvement had the highest agreement, while involvement of health care manager or administrator had the lowest. There were no significant differences in proportion of agreement across dyad types.

Agreement on engagement approaches ranged from 50.0 to 81.3%. Informal conversations had the highest proportion of agreement. Researchers attending knowledge user meetings had the lowest levels of agreement (50.0%). There were significant differences in the proportion of agreement across dyad types for one variable: reimbursement of expenses for knowledge users (*p* < 0.05), with NPI-researcher dyads having higher agreement than NPI-KU dyads.

**Table 4:** Proportion of agreement on individual variables

Variable	Full sample (*n* = 53)	Proportion of agreement, *n* (%)		
		NPI-team researcher dyads (*n* = 30)	NPI-KU dyads (*n* = 23)	Pearson Chi-Square (*p* value)
*Knowledge users engaged in the project (n* *= 53)*				
Industry representative	52 (98.1)	29 (96.7)	23 (100.0)	0.78 (0.38)
Community member	43 (81.1)	24 (80.0)	19 (82.6)	0.06 (0.81)
Health professional	42 (79.2)	22 (73.3)	20 (87.0)	1.47 (0.23)
Person with lived experience of a health condition	41 (77.4)	26 (86.7)	15 (65.4)	3.42 (0.06)
Health research funding organization representative	38 (71.7)	23 (76.7)	15 (65.2)	0.84 (0.36)
Community organization representative	38 (71.7)	20 (66.7)	18 (78.3)	0.86 (0.35)
Health professional organization representative	37 (69.8)	22 (73.3)	15 (65.2)	0.41 (0.52)
Health system decision or policy maker	28 (52.8)	19 (63.3)	9 (39.1)	3.06 (0.08)
Healthcare manager or administrator	25 (47.2)	16 (53.3)	9 (39.1)	1.05 (0.31)
*Approaches used to engage knowledge users in the project (n* *= 48)*				
Informal conversations	39 (81.3)	21 (80.8)	18 (81.8)	0.01 (0.93)
Formal in-person research project meeting	38 (79.2)	21 (80.8)	17 (77.3)	0.09 (0.76)
Distribution of study documents	35 (72.9)	17 (65.4)	18 (81.8)	1.63 (0.20)
Development of formal documentation of processes	34 (70.8)	21 (80.8)	13 (59.1)	2.71 (0.10)
Sharing of research funds with knowledge user organization	34 (70.8)	20 (76.9)	14 (63.6)	1.01 (0.31)
Provision of social opportunities	33 (68.8)	19 (73.1)	14 (63.6)	0.49 (0.48)
Provision of training opportunities	33 (68.8)	20 (76.9)	13 (59.1)	1.76 (0.18)
Formal online research project meeting	32 (66.7)	18 (69.2)	14 (63.6)	0.17 (0.68)
Shared electronic space	31 (64.6)	18 (69.2)	13 (59.1)	0.54 (0.46)
Honorarium for knowledge users	29 (60.4)	18 (69.2)	11 (50.0)	1.84 (0.18)
Establishment of formal working groups	27 (56.3)	13 (50.0)	14 (63.6)	0.90 (0.34)
Formal updates or newsletters about the research project	26 (54.2)	15 (57.7)	11 (50.0)	0.28 (0.59)
Reimbursement of expenses for knowledge users	25 (52.1)	19 (73.1)	6 (27.3)	**10.0 (< 0.01)**
Researchers attending knowledge user meetings or events	24 (50.0)	14 (53.8)	10 (45.5)	0.34 (0.56)

Bold indicates statistical significance

## Discussion

This is the first analysis we are aware of quantitatively comparing responses from individuals working on the same partnered health research project about characteristics at the level of the project that would be shared among individuals. The major finding from this analysis was the overall low level of agreement among respondents on questionnaire variables asking about practices in partnered Canadian health research projects between 2011 and 2019. These low levels of response agreement did not meet established methodological standards for acceptable agreement. This trend was consistent regardless of the roles compared (though NPIs and researchers consistently had higher agreement rates than NPIs and knowledge users), question, or variable content. The observed low levels of agreement were not related to the length of time respondents were required to recall the project, as there were no differences in mean agreement level by project funding year block. Variable-level agreement ranged between 47% and 98%, and there were no clear patterns in agreement on specific types of variables.

We are not aware of any studies with comparable data sets with which to compare the present analysis. We were surprised at that lack of agreement about partnering practices. The extensive variation in responses we observed may be due to a few factors. For example, there were a high number of response options available to represent a comprehensive range of knowledge user roles who could be represented on a project. It is possible that teams may not have focused extensively on titles, and therefore, these distinctions were not a primary focus of the work. Fewer categories of response variable options may have resulted in greater agreement. Differences may also be due to variations in communication across teams related to specific terms or activities used, and could have affected how participants responded. Regardless of the source of the variation that led to low agreement, the findings suggest interpreting NPI responses with caution, as they may not be verified by team members.

While this study is affected by the same limitations that affect all survey research and are limited by those who respond, these findings have several implications for the continued study and practice of partnered health research. Most critically, the findings highlight the limitations of self-report recall data and the caution that must be used in interpreting results. Our ongoing analyses of these projects will need to account for the multiple and varying practices reported. Moving forward, prospective documentation of partnered research practices offers the greatest potential to overcome the limitations of recall-based retrospective analyses. Development of tools and resources to assist teams in real-time documentation and tracking of partnership practices will enhance rigor and facilitate advanced study of the factors influencing successful partnership and impact of partnered health research. In addition, enhanced reporting standards can also support transparency regarding which partners are engaged and what strategies are used in a study. As we did not compare within-team responses for the perceived effects variables, future analyses may explore team variations on the perceived value of involving knowledge users as partners in health research. We also plan to conduct additional analyses of the present data exploring relationships between partnering practices and perceived effects of involving knowledge users in the eligible project. The present study is an important methodological analysis to inform project-level data processing decisions to ensure assumptions of independence. We acknowledge that despite extensive pilot testing, some respondents might have interpreted response options differently, which could have influenced agreement findings. A small number of “I don’t know” responses could not be included in concordance calculations. These likely reflect uncertainty or limited awareness of project activities rather than substantive disagreement.

## Conclusions

Low agreement about partnership practice characteristics between NPIs, other team researchers and knowledge users from the same Canadian partnered health research projects may be due in part to question design, retrospective recall, and/or shared understanding across teams, but has conceptual implications for how team members may differentially experience partnered research. Development of tools to support prospective documentation of partnering practices could strengthen both practice and study of partnered health research.

## Data Availability

The datasets used may be available from the corresponding author on reasonable request.

## Abbreviations

NPI: Nominated principal investigator
KU: Knowledge user
